# The Role of Optical Radiations in Skin Cancer

**DOI:** 10.1155/2013/842359

**Published:** 2013-04-24

**Authors:** Fabrizio Ayala, Marco Palla, Rossella Di Trolio, Nicola Mozzillo, Paolo A. Ascierto

**Affiliations:** Department of Melanoma, National Cancer Institute Pascale Foundation, Via Mariano Semmola, 80131 Naples, Italy

## Abstract

*Purpose*. Electromagnetic radiation with wavelength in the range 100 nm to 1 mm is known as optical radiation and includes ultraviolet radiation, the visible spectrum, and infrared radiation. The deleterious short- and long-term biological effects of ultraviolet radiation, including melanoma and other skin cancers, are well recognized. Infrared radiation may also have damaging biological effects. *Methods*. The objective of this review was to assess the literature over the last 15 years and to summarize correlations between exposure to optical radiation and the risk of melanoma and other cancers. *Results*. There is a clear correlation between exposure to UV radiation and the development of skin cancer. Most importantly, a strong association between artificial UV radiation exposure, for example, tanning devices, and the risk of melanoma and squamous cell carcinoma has been clearly demonstrated. There is no clear evidence that exposure to IR and laser radiation may increase the risk of skin cancer, although negative health effects have been observed. *Conclusions*. Preventative strategies that involve provision of public information highlighting the risks associated with exposure to sunlight remain important. In addition, precautionary measures that discourage exposure to tanning appliances are required, as is legislation to prevent their use during childhood.

## 1. Introduction

**Figure 1:**
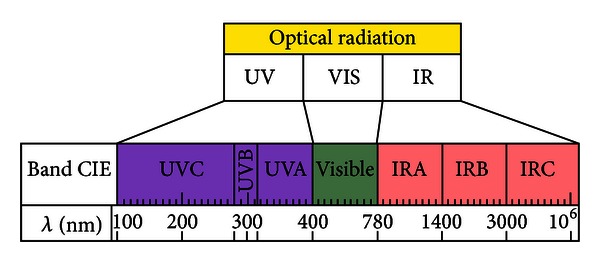
Wavelenghts of the main optical radiations.

Electromagnetic radiation with wavelength (*λ*) in the range 100 nm to 1 mm is known as optical radiation and includes ultraviolet radiation (UV; 100–400 nm), through the visible spectrum (380–780 nm) to infrared radiation (IR; 9780 nm–1 mm) (Figure 1) [1]. UV radiation is subdivided into three regions: UVC (100–280 nm), UVB (280–315 nm), and UVA (315–400), with minimal superimposition over the visible band in the range of 380–400 nm. IR radiation is also further divided into IRA (780–1400 nm), IRB (1400–3000 nm), and IRC (3000 nm–1 mm). These spectral bands, as defined by the International Commission on Illumination (CIE) in 1987 [2], represent the starting point for this consideration of the biologic effects of optical radiation.

### 1.1. Ultraviolet Radiation

Our planet is subjected to a solar radiation of about 1350 W/m^2^, although in reality only around 900 W/m^2^ reaches the Earth's surface because of the reflective effect of the stratosphere. Of this amount, the UV component constitutes a limited fraction (around 5%), since sunlight also consists of visible and infrared bands. The maximum UV radiation measured at ground level is 70 W/m^2^ (4200 J/min) of UVA, 2.5 W/m^2^(150 J/min) of UVB, and almost no UVC [3]. 

To have a better understanding of the characteristics of UV radiations that reach the Earth's surface, we should consider that at noon on a sunny day along the Mediterranean coast, the solar spectrum contains 95-96% UVA and 4-5% of UVB. The intensity of these radiations varies with time and location and is dependent on a range of factors, including hour of the day, season, latitude, altitude, weather, and degree of reflection. Taking these factors into account, the presumed dose of UV that reaches our body at a particular hour, day, and place can be evaluated as an index that is expressed on a scale of 1 to 10 [4]. This UV index is an indicator of the irradiance to the ground on a flat surface and can be used to predict how long people can safely stay exposed to the sun's radiation without deleterious biological effects. 

The damaging effects of UV radiation from the sun can be both short term and long term. Some of the adverse effects that are apparent after just a few hours of sunlight exposure (e.g., skin redness and burning) are due to the release of substances that cause vasodilation and erythema. Long-term effects include accelerated ageing (photoaging) of the skin, with a loss of elasticity and blotchy appearance, the onset of various skin tumours, cataracts, and also immunodepressive effects.

Cutaneous absorption of UV radiation is limited to the epidermis at wavelengths below 290 nm, while approximately 10% reaches the dermis in the range of 290–320 nm. About 50% of UV radiation reaches the cutaneous layer at wavelengths higher than 320 nm, meaning UVA is able to penetrate deeper into the skin than UVB. Since UVA also constitutes most of the UV spectrum reaching the Earth's surface, more UVA than UVB reaches the basal layers of the epidermis where keratinocytic stem cells and melanocytes are located [5]. 

Many studies have confirmed the mutagenic property of UV radiation [5–11]. UVB rays are carcinogenic agents that are directly absorbed by DNA and cause direct damage. This typically includes the formation of cyclobutane pyrimidine (CPD) dimers and 6-4 photoproducts (6-4P). Mutations induced by UVB are conversions such as C→T and CC→TT, commonly named the “UVB fingerprint” or “UVB signature.” UVB can also induce the formation of singlet oxygen (O^2−^), an extremely reactive oxidative compound that can indirectly damage DNA [12]. A recent study also suggested that C→T conversion can be induced by UVA [13]. 

Unlike UVB, UVA is not absorbed by DNA and so has no direct effect. Instead, UVA indirectly induces damage to DNA through the absorption of photons by other cell structures (chromophores) and the subsequent formation of oxygen reactive species (singlet oxygen and hydrogen peroxide). These principally react with guanine, thereby inducing DNA mutations. This damage is characterized by T→G conversions, known as “UVA fingerprint” or “UVA signature” mutations [14].

UVA and UVB cause cellular damage through different mechanisms [15, 16], although both act on expression of P53 and bcl-2 proteins that are involved in the regulation of apoptosis induced by UV radiation [17–20]. In fact, mutations in the P53 gene have been noticed both in basal cell carcinoma (BCC) [21, 22] and in squamous cell carcinoma (SCC) [23, 24]. Several studies have proven a pathogenetic correlation between UV radiation and skin cancer [25–29].

### 1.2. Infrared Radiation and Lasers

The International Commission on Nonionizing Radiation Protection (ICNIRP) offers limited information on trends in human exposure to IR radiations [30]. In recent years though, new types of IR heating devices, for example, dish warmers, infrared heating boxes (known as IR sauna) have been introduced for domestic use. Very little is known about the biologic effects of IR radiation, even though skin has considerable exposure to both natural and artificial sources. Epidemiologic and clinical data suggest that IR radiation is involved in the process of premature skin ageing and carcinogenesis, indicating that IR exposure is not entirely safe [31, 32]. It is worth noting that the damaging effects of RI radiation on crystalline lens through the action of heat in the iris are well recognized (e.g., in the high prevalence of cataracts observed in glass blowers).

The acronym L.A.S.E.R. (Light Amplification by Stimulated Emission of Radiation) refers to electromagnetic nonionizing radiations. Unlike ionizing radiations, the energy of nonionizing ones is not sufficient to ionize atoms and molecules by modifying bonds. However, they can break chemical bonds by means of photochemical reactions.

## 2. Materials and Methods

Data in this review have been extracted from reports and studies from the major international professional bodies and committees, including the International Commission on Nonionizing Radiation Protection (ICNIRP), the International Commission on Illumination (CIE), the World Health Organization (WHO), the INTERSUN Programme, the International Agency for Research on Cancer (IARC), the US. Environmental Protection Agency SunWise Program, the National Weather Service-Climate Prediction Center (NWS-CPC), the United Nations Environment Programme (UNEP), the World Meteorologic Organization (OMM), and the Electrotechnical International Committee.

These data have been complemented by a systematic literature review, in which various combinations of keywords have been used to search MEDLINE and identify relevant publications. These keywords consisted of ultraviolet radiation, UVR, infrared radiation, IR, cancer, tumor, skin cancer, melanoma, basal cell carcinoma, BCC, squamous cell carcinoma, SCC, sun tanning, sunburn, solaria, sunlamp, sunbed, artificial UV, laser, Nd:YAG, diode, Alexandrite, carbon dioxide, ruby, erbium:YAG, pulsed dye, and argon.

## 3. Results

### 3.1. Solar Exposure

Table 1 summarizes the principal studies that have investigated the association between UV radiation and skin cancer [33–43]. In many epidemiological studies, exposure to UV solar radiations has been recognized as the main environmental or behavioural cause for the appearance of melanoma. Although a dose-response type relationship between UV radiation exposure and the risk of melanoma is not always demonstrable because of confounding patient-specific variables, such as phototype and tendency to develop moles [41–44], excessive cumulative solar exposure (total lifetime hours) is well proven as the main causal factor in the pathogenesis of melanoma [45]. In particular, there is a strong correlation between the onset of melanoma and intense intermittent exposures or number of sunburn episodes. The relative risk of melanoma is significantly increased if these events happen during childhood and adolescence rather than in adult life (odds ratio (OR) 4.3 [1.7–11.1]) [35]. 

The pattern of exposure to solar radiations is a key determining factor in the occurrence of melanoma [46]. Most studies confirm a direct correlation between recreational or intermittent sun exposure and melanoma onset. For instance, in a meta-analysis of 29 case-control studies which assessed incident melanoma, sun exposure, and sunburn, Mark Elwood and Jopson (1997) reported an OR of 1.71 associated with intermittent solar exposure [34]. Conversely, an inverse correlation (i.e., a protective effect) was shown for heavy occupational exposure (OR 0.86). Another meta-analysis that included 57 studies also showed a protective effect of continuous exposure, especially in people who easily tan and rarely burn [47]. Indeed, many studies confirm the protective effect of continuous solar exposure during childhood and adolescence, particularly in individuals with phototype III-IV [33, 37], while excessive exposures, especially in people with fair skin and in the first 10 years of life, is associated with an increased risk of melanoma in later life [41, 48, 49]. Interestingly, some studies have indicated that artificially induced suntan, for example, before holidays/increased exposure to sunlight, has minimal to no protection against DNA damage [50–52].

Recent epidemiological studies have also demonstrated that solar UV radiations have a role in the onset of melanoma of the conjunctiva and iris (ocular melanoma), with pathogenetic mechanisms similar to those of cutaneous melanoma (OR 3.5 [1.2–8.9]) [53, 54]. The frequent confinement to the lower area of the iris confirms the role of UV radiations [55]. There is only a limited correlation between exposure to UV solar radiations and melanoma of the ciliary body and choroid, ocular areas not directly exposed to sunlight (OR 1.1 [0.7–1.6]) [56].

In addition to melanoma, both BCC and SCC are associated with UV radiation exposure. BCC is doubtless the most frequent skin cancer and UV rays represent the main cause of its onset [57]. The pattern of intermittent solar exposure and high doses of UV during childhood are more apparent in patients affected by BCC [37, 44, 45], whereas SCC is more strongly correlated with high doses of total or chronic-working exposures [44, 45]. 

### 3.2. Artificial UV Exposure: Sunbeds and Sunlamps

Since 2003-2004, when the US National Toxicology Program report on carcinogenesis recognized and classified total UV radiations as a carcinogenic agent for man [59], attention has been focused on the possible correlation between artificial exposure to UV radiations and skin cancer. As a result, in 2005, the IARC asked a group of experts to conduct a meta-analysis of studies assessing the correlation between artificial UV exposure and skin cancers. Their report, published in 2006, represents the most up-to-date document on this topic [58]. 

The results of their meta-analysis, which identified 19 studies with estimates of the relative risk (RR) for cutaneous melanoma associated with exposure to tanning appliances, are summarized in Table 2. The RR of melanoma associated with use of an indoor tanning facility was 1.15 (1.00–1.31). When the analysis was narrowed to include only the ten population-based case-control (*n* = 9) and cohort (*n* = 1) studies (i.e., excluding hospital-based studies), RR was 1.17 (0.96–1.42). When first exposure in youth (before age 35 years) was analyzed (7 studies), a significant 75% increase in risk was observed (RR 1.75 [1.35–2.26]). For this reason, a recent report from IARC reclassified these devices as being among those that emit radiations carcinogenic to humans (Group 1) [60]. No strong evidence concerning a dose-response relationship between artificial UV exposure and risk of melanoma was identified.

The IARC also assessed the relationship between SCC and BCC and exposure to artificial UV radiation. Indoor tanning was shown to significantly increase the risk of SCC (three studies; RR 2.25 [1.08–4.70]), but no effect was observed for BCC (four studies; RR 1.03 [0.56–1.90]) [60]. 

### 3.3. UVB Devices for Phototherapy

Artificial UVB devices are used to treat many cutaneous diseases, in particular psoriasis. This has typically involved the use of wide-band UVB, although narrow-band devices have also recently been shown to be beneficial. Studies have shown no significant relation between the use of UVB devices for phototherapy and the incidence of melanoma, BCC, or SCC [61–68]. Nevertheless, although these data may seem reassuring, they cannot exclude the possibility of an increased tumour risk in patients receiving high doses of UVB [69].

### 3.4. PUVA Therapy

PUVA therapy involves the combination of psoralen, a light-sensitizing medication, with UVA and is used in the treatment of psoriasis and other skin conditions. Overall, there appears to be good evidence that PUVA increases the risk of SCC, although it can be difficult to identify causality since patients often receive several other potentially carcinogenic treatments. Two cohort studies have reported an association between PUVA therapy and skin cancer: one that included 4799 patients in Sweden [70], and a second that included 1380 patients in the USA [71]. In the Swedish study, the RR of SCC onset was 5.6 (4.4–7.1) in men and 3.6 (2.1–5.8) in women. In the US cohort, approximately one-quarter of patients who had received more than 2000 J/cm^2^ developed SCC. A meta-analysis by the same group reported that patients exposed to high doses of PUVA (more than 200 treatments or more than 2000 J/cm^2^) had a risk 14 times higher than those treated with less than 100 sessions or exposed to less than 1000 J/cm^2^ [72]. Another recent 30-year prospective study showed that receiving between 350 and 450 PUVA treatments had an RR of 6.0 (4.4–8.2) for SCC compared with less than 50 treatments. However, even high-dose exposure did not increase BCC risk [73]. 

The risk of melanoma onset associated with PUVA is more controversial. The American cohort study [71] reported an increased risk of melanoma, with patients exposed to more than 200 treatments compared to lower doses having an almost threefold greater risk (RR 2.9 [1.3–6.4]). Moreover, this risk increased over time, with an incident relative risk of 5.0 (1.6–15.5) among patients after >15 years followup (versus <15 years). In the Swedish cohort, an increased risk for melanoma was not observed. Since the Swedish study is both larger and has a longer period of followup (on average 16 years), data obtained from this cohort are the more convincing. 

### 3.5. Exposure to Infrared and Laser Radiations

As with UV radiation, extended exposure (15–20 years) to IR radiation can induce actinic keratosis, a possible precursor to *in situ* or invasive carcinomas. It is also known that prolonged exposure of the skin to heat induces particular changes known as warmer erythema, heat dermatitis, or *erythema ab igne*. However, there are only limited data on the topic of IR radiation and cancers (in particular of the skin), with most being case reports of tumours arising secondarily to *erythema ab igne *after many years [73–87]. 

At the present moment there are no studies that demonstrate potential carcinogenicity of laser devices, although this could depend on the relative rare occurrence of exposure to laser beams. There are case reports in the literature concerning malignant tumours arising from benign lesions after prolonged laser treatments. However, the possibility of diagnostic error before laser therapy cannot be excluded in these rare cases [88–97].

## 4. Conclusions

Our review of the literature shows a clear association between UV radiation exposure and increased risk of melanoma and other skin cancers. However, there is no clear evidence of any relationship between skin cancer and IR or laser radiations. 

Preventative strategies that include public information campaigns to highlight the risks associated with exposure to sunlight, including the degree of exposure that is considered acceptable with regard to health and identifying patient types most at risk, remain important. The use of artificial tanning devices requires special consideration. It is now clear that there is a strong association between these devices and the risk of melanoma and SCC. As such, precautionary measures that discourage exposure to tanning appliances, especially among younger people, are required, as is legislation to prevent their use during childhood. Public health initiatives comparative to those that have successfully targeted cigarette smoking are now needed to limit recreational exposure to artificial UV sources.

## Figures and Tables

**Table 1:** Main published studies on association between solar UV exposure and the risk of melanoma, basal cell and squamous cell, carcinoma onset.

Reference	Type of exposure	Epidemiological index Odds ratio (95% CI)	Comment
Melanoma			

White et al. (1994) [33]	Chronic	0.3 (0.16–0.59)	Exposure aged 2–20 years

Mark Elwood and Jopson (1997) [34]	Chronic (occupational)	0.86 (0.77–0.96)	Meta-analysis of 29 studies
	Intermittent	1.71 (1.54–1.90)	
	Total	1.18 (1.02–1.38)	

Autier and Doré(1998) [35]	>1 year tropical or subtropical area	4.3 (1.7–11.1)	Exposure aged <10 years
	>1 year tropical or subtropical area	4.1 (1.3–13.4)	Exposure in adolescence or adulthood

Walter et al. (1999) [36]	Chronic	0.67 (0.52–0.85)	Exposure aged <18 years
	Intermittent	1.67 (1.31–2.12)	

Kaskel et al. (2001) [37]	Chronic	0.3 (0.1–1.1)	Exposure aged <12 years
	Intermittent	2.4 (1.2–4.9)	

Whiteman et al. (2006) [38]	Chronic	2.49 (1.12–5.54)	Head and neck
	Intermittent	0.38 (0.17–0.83)	

Kricker et al. (2007) [39]	Chronic	1.03	Multiple versus single melanoma
	Intermittent (beach)	1.85	
	Intermittent (recreational)	1.38	

Nagore et al.(2010)[40]	Chronic (<20 years)	0.6 (0.3–1.3)	Age at diagnosis >60 years
	Chronic (>20 years)	2.1 (1.1–4.0)	

Basal cell (BCC) and squamous cell carcinoma (SCC)			

Armstrong and Kricker (2001) [41]	*BCC*:		Meta-analysis
	Chronic	1.19 (1.07–1.32)	
	Intermittent	1.38 (1.24–1.54)	
	Total	0.98 (0.68–1.41)	
	*SCC*:		
	Chronic	1.64 (1.26–2.13)	
	Intermittent	0.91 (0.68–1.22)	
	Total	1.53 (1.02–2.27)	

Zanetti et al. (2006) [42]	*BCC*:	
	Chronic (occupational)	1.2 (0.70–2.13)
	Intermittent	1.3 (0.72–2.39)
	Total	1.7 (0.97–3.03)
	*SCC*:	
	Chronic (occupational)	2.2 (1.13–4.08)
	Intermittent	0.6 (0.29–1.21)
	Total	1.8 (0.95–3.32)

Han et al. (2006) [43]	BCC total	1.95 (1.34–2.83)
	SCC total	1.97 (1.37–2.85)

**Table 2:** Results of IARC meta-analysis of studies on the correlation between sun lamp exposure and the risk of melanoma, basal cell carcinoma, and squamous cell carcinoma onset [58].

	Studies (*n*)	Relative risk (95% CI)	Heterogeneity *P* value *χ* ^2^
Melanoma			
Sun lamp exposure	19	1.15 (1.00–1.31)	0.013
First exposure in young age	7	1.75 (1.35–2.26)	0.55
Past exposure	5	1.49 (0.93–2.38)	0.018
Recent exposure	5	1.10 (0.76–1.60)	0,05625
Basal cell carcinoma			
Sun lamp exposure	4	1.03 (0.56–1.90)	0.06
Squamous cell carcinoma			
Sun lamp exposure	3	2.25 (1.08–4.70)	0.10
